# Periosteum for Root Coverage in an Isolated Gingival Recession as an Autogenous Graft: A Case Report

**DOI:** 10.7759/cureus.60207

**Published:** 2024-05-13

**Authors:** Mohanasatheesh S, Dheraj Sayan, Ranjith Mari, Anitha Balaji, Mohan Valiathan

**Affiliations:** 1 Periodontics, Sree Balaji Dental College and Hospital, Chennai, IND; 2 Periodontics, Sree Balaji Dental College and Hospital, chennai, IND; 3 Periodontics, Sree Balaji dental College and Hospital, chennai, IND

**Keywords:** isolated gingival recession, autograft, root coverage, leukocyte platelet rich fibrin (l-prf), periosteum

## Abstract

In periodontal care, where patient results are crucial in guiding the development of surgical techniques, gingival recession management is a critical issue. The periosteum eversion technique (PET) emerges as a modern strategy that leverages the intrinsic regenerative capabilities of the periosteum to attain root coverage. A detailed case study showcases the effectiveness of PET in managing a Miller Class I gingival recession alongside an adjunctive platelet-rich fibrin (PRF) procedure. This approach entailed the deliberate elevation and eversion of the periosteal flap to encompass the recession area, securing it meticulously through suturing. Across a six-month observation period, this method exhibited successful root coverage, augmentation of keratinized tissue, and enhanced patient comfort, as reported, with no significant complications observed. These outcomes provide support for the incorporation of PET into standard periodontal protocols, underscoring its capacity to reshape the treatment landscape for gingival recession.

## Introduction

The exposing of the root surface from the gingival edge migrating to the cementoenamel junction (CEJ) is known as gingival recession. It could be connected to one or more tooth surfaces and could be localized or widespread (Kassab and Cohen 2003) [1]. This condition has the potential to give rise to aesthetic anxieties, heightened sensitivity, and various other associated intricacies [2]. Gingival recession can be localized (affecting a single tooth) or generalized (impacting multiple teeth). Factors like aging, anatomy, and trauma contribute to its development. It is classified based on Miller’s classification, which considers the level of recession and interdental tissue damage [3].

The etiological factors underlying gingival recession include periodontal diseases, traumatic injuries, and prosthetic stimuli. Risk factors that predispose individuals to gingival recession are observed in the periodontal biotype, where even minimal brushing force can result in recession, particularly in isolated defects such as those found in the maxillary canine. These recessions may serve as reservoirs for plaque accumulation, thus exacerbating the condition. One of the main causes of gingival recession has been identified as improper teeth-brushing practices that result in mechanical damage [4]. Root covering is indicated for the treatment of root caries or cervical abrasions as well as for esthetic reasons to lessen root sensitivity, as per the American Academy of Periodontology's position paper from 1996 on mucogingival therapy [5]. Various methods for root coverage have been employed with varying levels of success. These include free gingival autografts, connective tissue grafts, coronally advanced flaps (CAFs), lateral pedicle flaps, and tunneling. Additionally, allogenic soft tissue substitutes, like acellular dermal matrix, and xenogenic soft tissue substitutes, such as Novamatrix, have also been utilized. Maintaining ideal gingival height and general dental health is still the primary objective of various therapeutic strategies, and biological regeneration level determines clinical success [6]. In 2005, Gaggl et al. introduced a novel technique called periosteum eversion/perioplasty [7]. This method involves using the underlying periosteum to manage recession defects in dental procedures. Since its inception, numerous case reports and clinical studies have demonstrated positive outcomes related to this approach. For the restoration of exposed root surfaces, the periosteum - a highly cellular connective tissue having abundant vascularity and the capacity for regeneration - acts as an autogenous graft. Its effectiveness is attributed to its ability to secrete vascular endothelial growth factor (VEGF), which endorses revascularization during the process of wound repair, the internal layer contains osteoblasts, and osteoprogenitor cells while the external layer consists of densely arranged collagen fibers, fibroblasts, and precursors [8]. At all stages of life, the cells within the periosteum retain the capacity to transform into fibroblasts, osteoblasts, chondrocytes, adipocytes, and skeletal myocytes, giving rise to various tissues, including cementum, periodontal ligament fibers, and bone. The periosteum is characterized by its rich vascular network and is often likened to the "umbilical cord of bone" [9]. Regarded as a covert protagonist, the periosteum significantly contributes to tissue repair and regeneration. One of the advantages of utilizing the periosteum for root coverage lies in its minimally invasive nature, resulting in fewer complications for the patient. This is achieved by utilizing the adjacent periosteum as a pedicle graft to effectively conceal the root surface leukocyte platelet-rich fibrin (l-PRF) capitalizes on the body’s innate healing capabilities, functioning as a valuable instrument in the realm of periodontal therapy. With its elevated levels of platelets, growth factors, and cytokines, platelet-rich fibrin speeds up soft tissue mending, diminishes inflammation, and stimulates regeneration. Healthcare providers frequently use l-PRF for tasks such as root coverage, enhancement of soft tissue, and enhancement of wound healing near dental implants. Data substantiates l-PRF as a supplementary component to traditional methodologies in management of the gingival recessions [10].

## Case presentation

**Figure 1:**
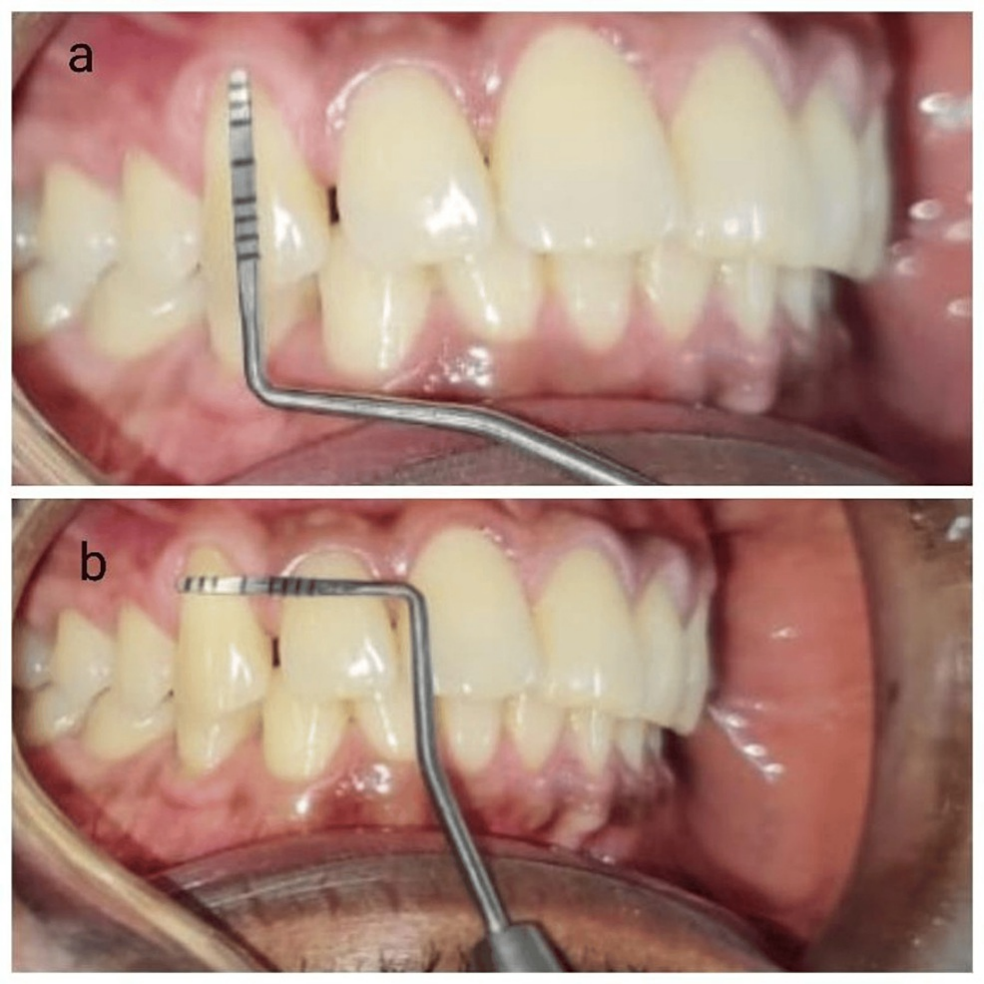
Preoperative clinical picture 1A: showing gingival recession 2 mm from the cementoenamel margin junction to the coronal gingival margin; 1B: the width of the recession can be appreciated

Gingival recession in the frontal maxillary area was the main complaint of a 34-year-old male patient. Upon intraoral inspection, the labial surface of the upper right canine (tooth 13) revealed a single Miller class I gingival recession, in accordance with the Fédération Dentaire Internationale (FDI) tooth numbering system. By means of a Goldman Fox, the recession depth was computed as 2 mm from the CEJ to the gingival edge (Figure 1). Following this, the patient received phase I intervention for basic periodontal therapy, which comprised root planning, scaling, and instructions on appropriate dental hygiene practices. The individual provided informed consent, and comprehensive details regarding the surgical procedure were communicated to the patient.

**Figure 2:**
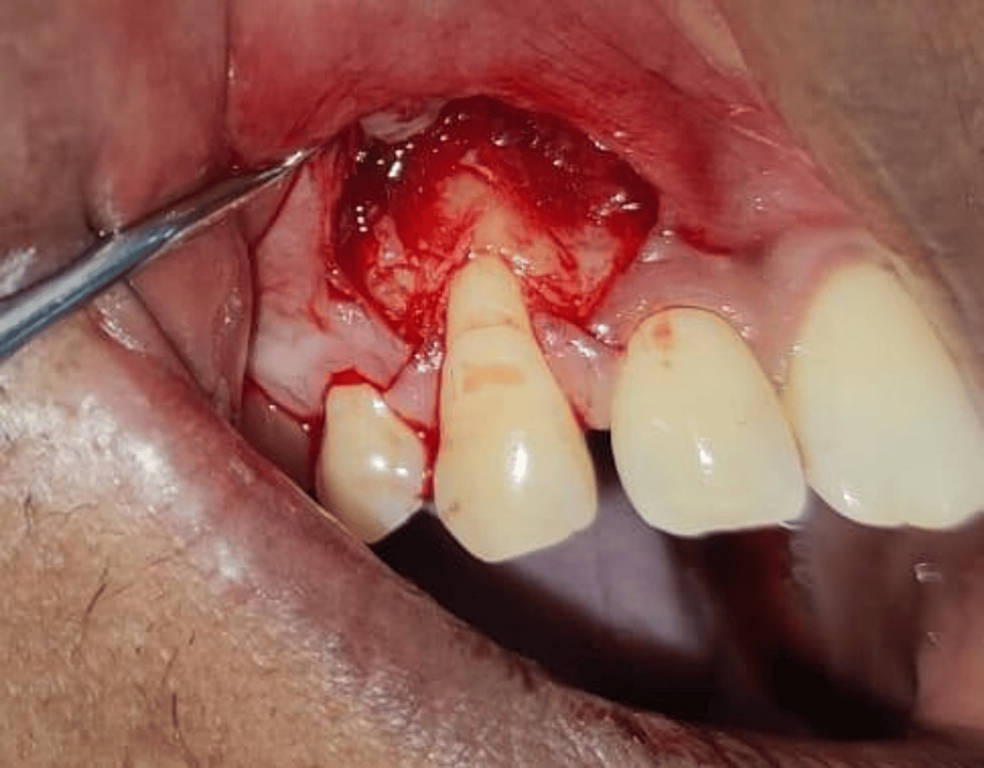
Partial thickness flap reflected

The patient underwent preliminary preparation involving the application of a 0.12% chlorhexidine mouthwash, adhering to standard preoperative procedures. Subsequently, an infiltration of 1:100000 lignocaine with adrenaline was administered. Local anesthesia was induced, and an incision was performed meticulously, with lateral vertical proximal incisions. The partial thickness flap was then skillfully reflected, exposing the root surface for thorough scaling, root planning (Figure 2), and conditioning with 24% ethylenediamine tetra-acetic acid for a duration of three minutes, followed by irrigation with a regular saline solution for 60 seconds.

**Figure 3:**
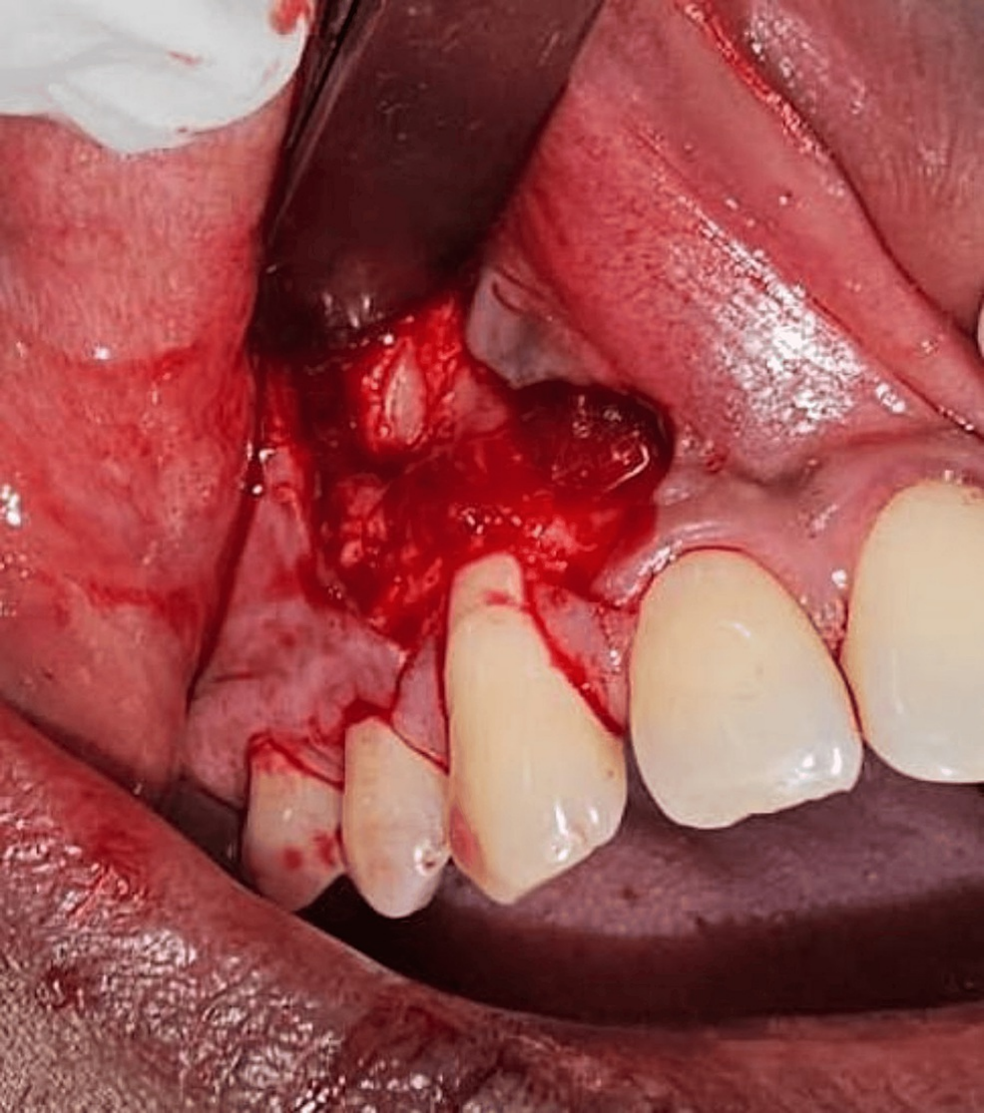
Periosteum incision given at the apical end

**Figure 4:**
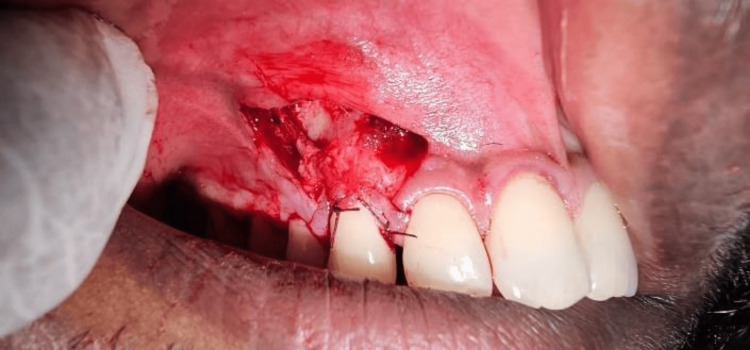
Periosteum being everted and adapted over the recession area with 5-0 vicryl suture

**Figure 5:**
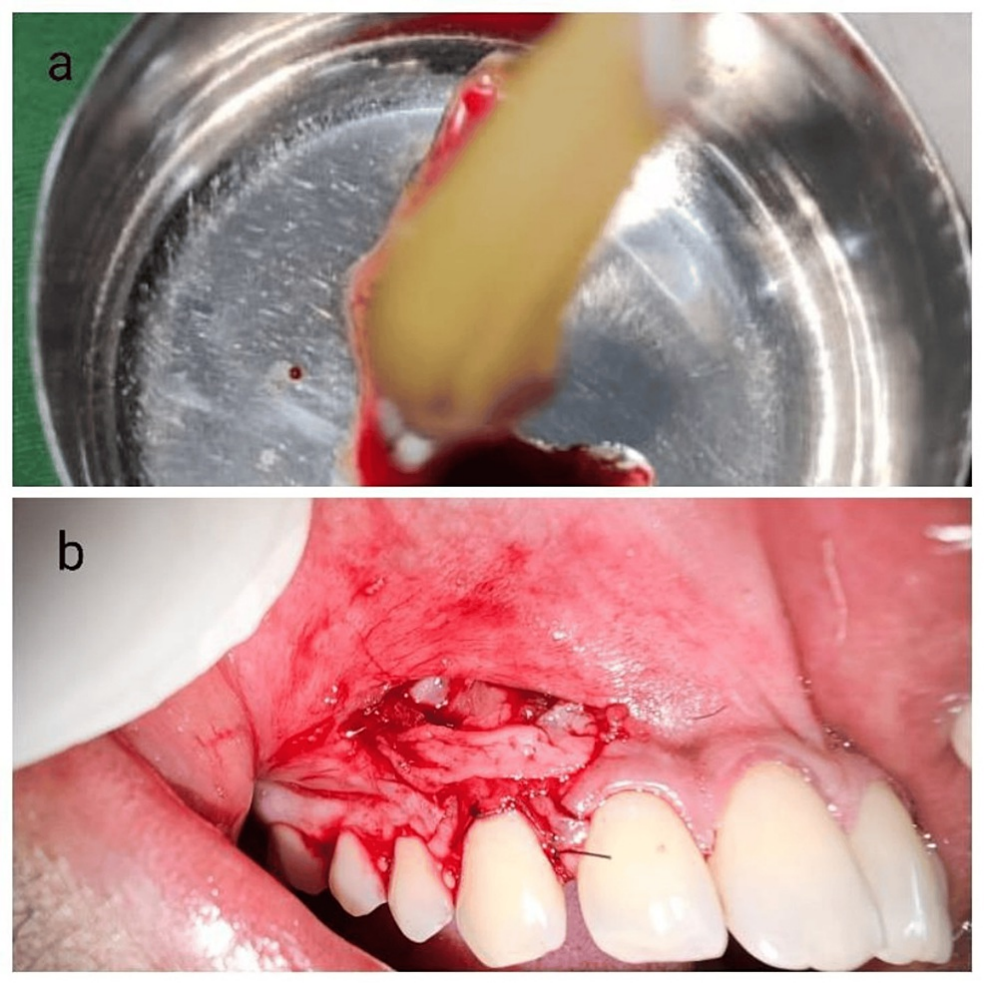
Adaptation of the l-prf membrane over the periosteum 5A: l-prf coagulum taken; 5B: l-prf placed over the periosteum l-prf: leukocyte platelet-rich fibrin

**Figure 6:**
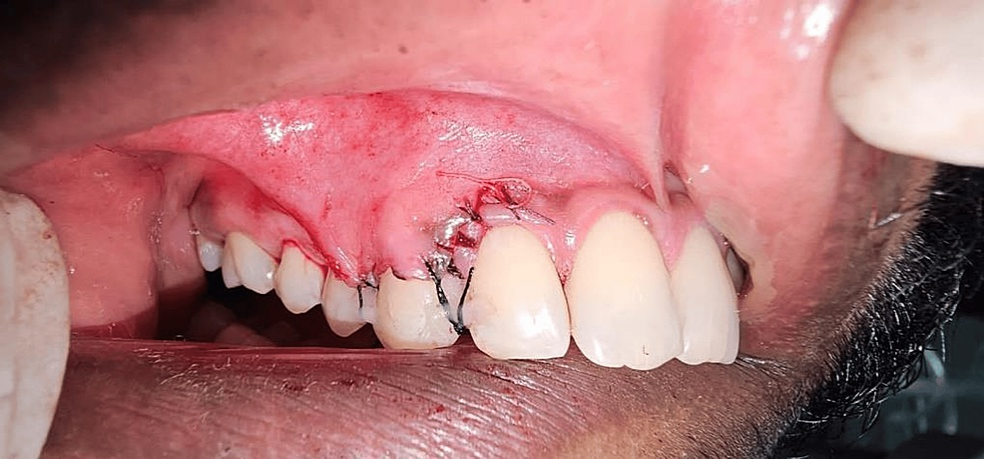
Flap coronally advanced covering l-prf and periosteum secured with 5-0 vicryl suture l-prf: leukocyte platelet-rich fibrin

To cut through the periosteum, a basal incision was made at the baseline. From there, the submucous connective tissue was detached and the associated gingiva's border was reached. The crestal periosteum pedicle was everted (Figure 3) and mobilized to cover the recessed area, securing it in place with 5-0 absorbable sutures (Figure 4). l-PRF (leukocyte platelet-rich fibrin) is placed on the periosteum (Figure 5). Additionally, Figure 6 shows that a coronal transposition of the mucoperiosteal flap was performed and sutured using 5-0 absorbable sutures.

The patient was told to take non-steroidal anti-inflammatory drugs (NSAIDs) and a course of antibiotics for five days. They were also told to use mouthwash containing chlorhexidine instead of brushing their teeth for a week. Following the first week, the patient was advised to continue using Chlorhexidine mouthwash and brush their teeth using the modified stillman technique. 

**Figure 7:**
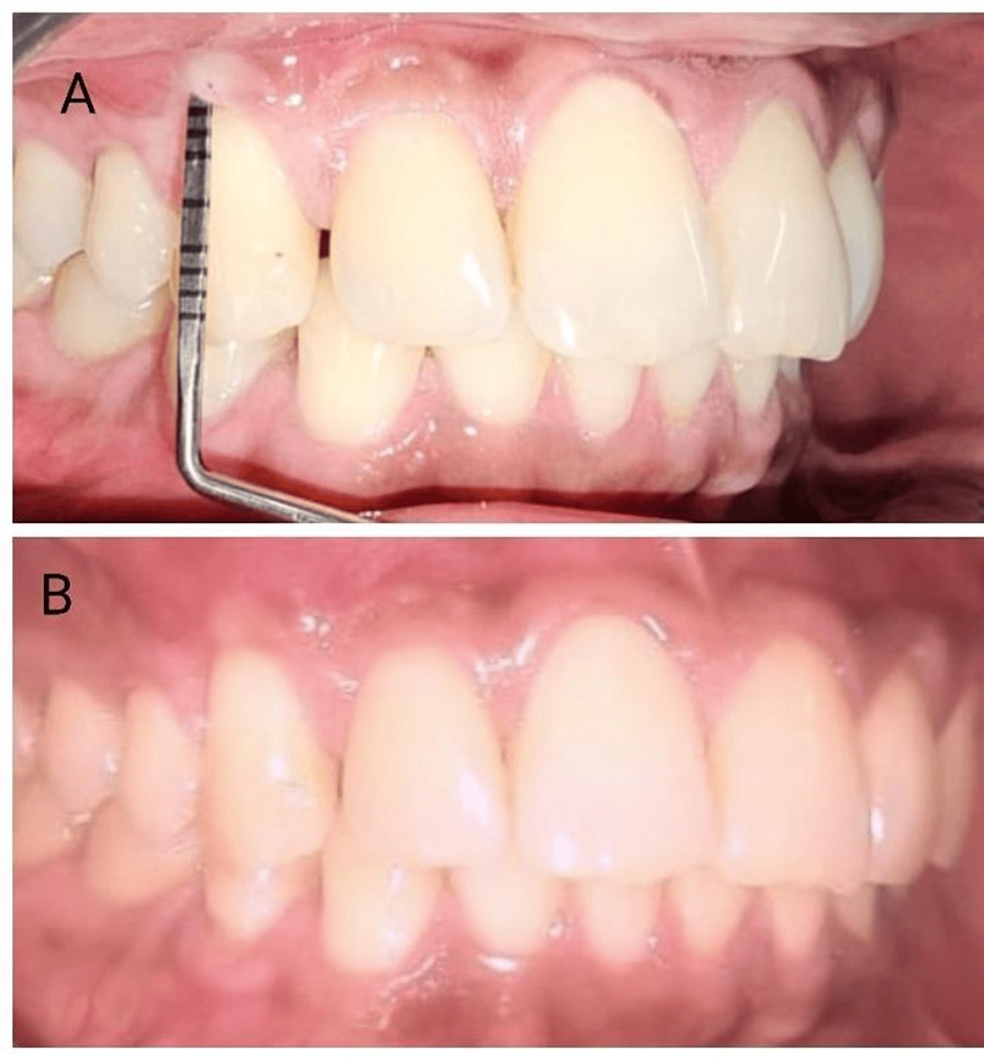
Three-month postoperative picture 6A: sulcus depth lesser than 1 mm after coverage; 6B: complete root coverage achieved

The patient was scheduled for weekly follow-up appointments during the postoperative month. At each visit, instructions on maintaining dental hygiene were provided and monitored. The healing process was smooth, and the patient expressed satisfaction. Subsequently, monthly routine follow-up calls were made. After three months, progressive adaptation and morphological resemblance were observed, resulting in 100% root coverage (Figure 7).

**Figure 8:**
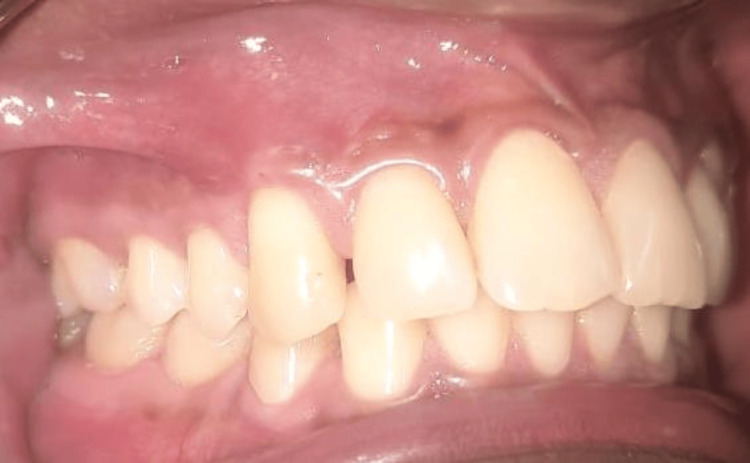
Six-month postop picture

Additionally, the patient achieved a probing depth of one millimeter. The patient has been re-called for six months for follow-up (Figure 8).

## Discussion

Research has demonstrated that periodontal deficiencies can be treated with connective tissue grafts using the periosteum as a barrier membrane (Lekovic et al. 1991) [11]. 

The periosteum eversion technique (PET) and the sub-pedicle connective tissue graft technique (SPCTGT) were compared for root treatment of gingival recessions in random split-mouth research by Singh et al. in 2019 [12]. The results showed that PET was more successful than SPCTGT. Greater improvements were seen by PET in the areas of attached gingiva width probing depth, keratinized gingiva width, and gingival recession depth.

Periosteal pedicle graft (PPG) paired with a coronally advanced flap showed a high mean root coverage (mRC) of 87.7% for specifically localized gingival recession abnormalities and 84.83% for numerous defects, in accordance with a systematic review meta-analysis by Mahajan et al. in a 2023 study. There was an overall rise in the width of keratinized gingiva (WKG) among all involved investigations in the PPG + CAF group. Patient satisfaction was reported to be better with PPG + CAF compared to other techniques like subepithelial connective tissue grafts and coronal advanced flaps. The study concluded that PPG + CAF is a better choice for managing gingival recession defects, with results comparable to conventional techniques [13].

According to a case series by Avinash and Grumoorthy (2021), periosteal eversion can effectively repair gingival recession before orthodontic correction, as evidenced by its ability to cover 75% of the denuded root surface after just six months [14].

The treatment outcomes for both the coronal advanced flap with a platelet-rich fibrin membrane as graft and the PET by means of the periosteum as graft were statistically nonsignificant, as per a relative study investigation carried out by Koel et al. in 2018 [15]. Specifically, the mean root coverage (RC) percentages for CAF + PRF and PET were 75.01% vs. 86.86% (probe method) and 61.112% vs. 83.971% (vernier method) at the six-month follow-up, with no significant difference.

PET is effective for treating gingival recession, supplying greater gingival height, decreased probing depth, and 100% root coverage. However, it requires postoperative care for optimal results and requires surgical skills due to tissue attachment. Patient satisfaction and complete epithelialization were observed, with weekly clinical follow-ups for the first month of post-surgery, in a case report by AK Singh et al. (2015) [16].

Our case report involved amalgamating LPRF with an adjuvant factor to enhance the modulation of the immune system and facilitate soft tissue wound healing. This method also aims to enhance the adaptability of the periosteum to the exposed surface through a series of proliferation and differentiation processes triggered by an apical incision. Conversely, the fibrous layer will adhere to the avascular tooth surface following the debridement of the root surface using 24% ethylenediaminetetraacetic acid (EDTA). The periosteal layer, when pedicled at the crestal level, ensures a continuous blood supply to the area. Furthermore, the progenitor cells surrounding the regions of root coverage play a crucial role in generating new attachments by producing cementum [16].

To the best of the author's knowledge, techniques involving periosteum eversion have not been documented for root coverage in isolated gingival recession specifically on the maxillary canine utilizing LPRF. Studies have indicated that LPRF contributes positively to the process of wound healing as opposed to regeneration, and it also holds significance in terms of patient-reported outcomes related to postoperative pain.

While connective tissue graft (CTG) is commonly accepted as the preferred method for root coverage procedures, in our situation, we encountered shallow and narrow recession accompanied by a Nordland Type 1 papilla [17]. Consequently, we opted to capitalize on the benefits of periosteum over periosteal pedicle graft as the periosteum eversion technique avoids the inherent retraction force of periosteum to its position especially in the maxillary teeth due to its elastic nature. L-prf synergistically acts as a slow-releasing growth factor and helps in increasing the thickness of the biotype.

Nevertheless, to evaluate this technique's effectiveness and cost-effectiveness, more research is necessary. Although additional research is imperative to unveil the complete potential and constraints of this method, our discoveries imply that it exhibits promise as a minimally invasive and efficacious approach to enhance gingival tissue regeneration and refine aesthetic outcomes in periodontal therapy.

## Conclusions

The combination of periosteum eversion and PRF creates a conducive environment that promotes bone regeneration and tissue healing during surgical procedures. Although initial findings are promising, more research is required to fully understand its capabilities and fine-tune its use in clinical settings.

The periosteum eversion technique, due to its distinct vascularized nature, offers several benefits such as accurate harvesting, absence of donor site complications, and reduced chances of infection, necrosis, and graft rejection. Importantly, in the context of the isolated recession defect in our case, we managed to achieve the maximum possible root coverage.
